# Determination of Sugars and Acids in Grape Must Using Miniaturized Near-Infrared Spectroscopy

**DOI:** 10.3390/s23115287

**Published:** 2023-06-02

**Authors:** Lucie Cornehl, Julius Krause, Xiaorong Zheng, Pascal Gauweiler, Florian Schwander, Reinhard Töpfer, Robin Gruna, Anna Kicherer

**Affiliations:** 1 Julius Kühn Institute (JKI), Federal Research Centre of Cultivated Plants, Institute for Grapevine Breeding Geilweilerhof, 76833 Siebeldingen, Germany; 2 Fraunhofer Institute of Optronics, System Technologies and Image Exploitation IOSB, 76131 Karlsruhe, Germany

**Keywords:** grapevine, NIRS, quality, ripening, harvest, precision viticulture, field phenotyping

## Abstract

An automatic determination of grape must ingredients during the harvesting process would support cellar logistics and enables an early termination of the harvest if quality parameters are not met. One of the most important quality-determining characteristics of grape must is its sugar and acid content. Among others, the sugars in particular determine the quality of the must and wine. Chiefly in wine cooperatives, in which a third of all German winegrowers are organized, these quality characteristics serve as the basis for payment. They are acquired upon delivery at the cellar of the cooperative or the winery and result in the acceptance or rejection of grapes and must. The whole process is very time-consuming and expensive, and sometimes grapes that do not meet the quality requirements for sweetness, acidity, or healthiness are destroyed or not used at all, which leads to economic loss. Near-infrared spectroscopy is now a widely used technique to detect a wide variety of ingredients in biological samples. In this study, a miniaturized semi-automated prototype apparatus with a near-infrared sensor and a flow cell was used to acquire spectra (1100 nm to 1350 nm) of grape must at defined temperatures. Data of must samples from four different red and white *Vitis vinifera* (L.) varieties were recorded throughout the whole growing season of 2021 in Rhineland Palatinate, Germany. Each sample consisted of 100 randomly sampled berries from the entire vineyard. The contents of the main sugars (glucose and fructose) and acids (malic acid and tartaric acid) were determined with high-performance liquid chromatography. Chemometric methods, using partial least-square regression and leave-one-out cross-validation, provided good estimates of both sugars (RMSEP = 6.06 g/L, *R*^2^ = 89.26%), as well as malic acid (RMSEP = 1.22 g/L, *R*^2^ = 91.10%). The coefficient of determination (*R*^2^) was comparable for glucose and fructose with 89.45% compared to 89.08%, respectively. Although tartaric acid was predictable for only two of the four varieties using near-infrared spectroscopy, calibration and validation for malic acid were accurate for all varieties in an equal extent like the sugars. These high prediction accuracies for the main quality determining grape must ingredients using this miniaturized prototype apparatus might enable an installation on a grape harvester in the future.

## 1. Introduction

The ripening process of grapes follows a three-part scheme, two growth cycles separated by a lag phase [1,2]. In the first growth phase, malic acid and tartaric acid are accumulated, thus increasing the acids content to its maximum. During the second growth period, onwards from véraison, sugar concentrations increase rapidly and acid contents begin to steadily decrease. While malic acid is mainly metabolized, tartaric acid is diluted by growth in this phase [3]. These growth phases depend highly on the weather and, thus, on the prevailing climatic conditions and their changes. It is already becoming apparent that the predicted climatic changes can lead to musts of lower quality and a more difficult vinification [4]. Due to rising temperatures and mild winters in the Northern Hemisphere, véraison tends to take place earlier in the year. As a consequence, ripening is in the warmest period of the year. This, combined with the predicted rising temperature [5], results in a separation of sugar and acid metabolisms [6]. Excessive sugar contents are possible, resulting in undesirable high alcohol contents. Furthermore, acid contents become far too low, due to concentration of sugars [7,8] and further metabolization of acids (especially malic acid) [6]. This impairment of the delicate sugar–acid balance is particularly disadvantageous for the production of high-quality wines, as the sugar, alcohol, and acid contents largely determine the taste of the wine.

In order to obtain a broader view of the maturity stage of the grapes, it is possible to carry out a laboratory analysis. In this case, most commonly Fourier-Transform infrared spectroscopy (FTIR) is used to measure various additional parameters, for example volatile acids, pH, or the content of yeast-usable nitrogen. However, this method is labour intense as clear must samples have to be prepared for the measurements in the laboratory. Additionally, specialized personal and equipment is required, and it is expensive and time-consuming. Such measurements of the quality attributes such as the sugars and acids contents can be carried out prior to harvest, but are then made again upon delivery in the cellar. The differences between these measurements show that accurate predictions of quality in the field is crucial and appropriate techniques are needed. This could be overcome through a sensor measuring important quality parameters in the tank of the grape harvester during the harvesting process. However, there is a lack of usable, inexpensive technology on the market, that collects these parameters fully automatically.

In vibrational spectroscopy, there is a so-called fingerprinting region. This region is below 1500 cm${}^{-1}$, where a highly complex spectrum is obtained that can be clearly assigned to an organic substance. However, working in such wavelength ranges so far requires expensive sensors intended for the scientific use. Often some sample preparation is necessary and such sensitive spectrometers are not sufficiently robust for use in the field. To overcome this, near-infrared spectroscopy (NIRS) can be used. This is a reliable, common, and widely used easy-to-operate technique, detecting infrared radiation between 760 nm and 2500 nm. The obtained spectra contain overtones and combination of vibrations, requiring chemometric methods for the content predictions. After calibration, results could be achieved quickly and easily, and excessive sample preparation could be circumvent.

Using diffuse reflectance spectra, the reflected and returning light of an illuminated sample would be recorded. Only a small proportion of the radiation entering the sample and directly interacting with the ingredients returns. Disturbing influences on such spectra are manifold and high coefficients of variation are reported when applying this technique [9]. For this type of measurement, a great deal of research and development is still needed before suitable solutions can be found. Currently, measurements in transmission mode are more suitable. There, a light beam is guided through the homogenized sample. As a result, in particular light scattering effects can be minimized and coefficients of variation can be reduced. A high proportion of the radiation interacts with the target molecules and a higher amount of information about them are included. This enables the construction of better models using chemometrics resulting in a higher predictability of ingredients in biological samples using NIRS. Some properties as well as substances in agricultural products could already be determined on- and in-line using this technique (for an overall overview see Huang et al. [10], especially Cozzolino et al. [9] for viticulture). Due to the strong absorption of water in this wavelength range NIRS is most often used to determine the moisture content of a sample, but also the determination of alcohols in beverages is completed. Moreover, a handheld digital refractometer using NIRS for the sugar determination in individual grapevine berries is already available on the market. However, no usable, reliable and inexpensive sensors using NIRS are available for measuring important quality parameters like sugars and acids before winemaking.

There are still some challenges to overcome. Grape must is highly variable and contains many substances influencing the quantification of metabolites of interest. The changing chemical matrix can have influences on the spectra and, thus, can affect the predictive capacity of this technique. González-Caballero et al. [11] and Fernández-Novales et al. [12] have recorded transmission spectra of white and red grape must of different grapevine varieties. However, they did not create the calibrations specifically for the variety and, thus, increased the variance in the dataset. A wide wavelength range, as it was chosen in the study of González-Caballero et al. [11], requires expensive spectrometers. The prediction of quality-determining compounds can be executed using smaller wavelength ranges, which was shown by Fernández-Novales et al. [12]. However, until now acids have been less predictable. Krause et al. [13] could show the attribution of sugars and acids to particular wavelength bands in the range of 1100 nm to 1160 nm and 1300 nm to 1350 nm, respectively. These should be taken into consideration to achieve precise results, especially for the acids contents prediction.

Reliable and robust models for must of several *Vitis vinifera* (L.) varieties are still needed. This study is about the prediction of the important quality determining parameters, main sugars and acids in different grape musts using miniaturized NIRS. Sample preparation was minimal and models were build using accurate reference values gained with high-performance liquid chromatography (HPLC).

## 2. Materials and Methods

### 2.1. Plant Material and Sampling

#### 2.1.1. Grapevine Farming Sites and Used *Vitis vinifera* (L.) Varieties

**Figure 1:**
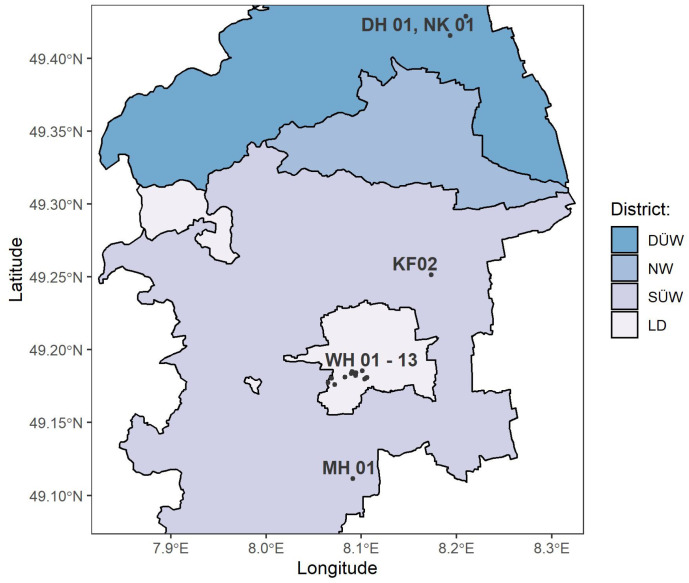
Map of the plots used in Germany, southern Rhineland Palatinate, districts of Bad Dürkheim an der Weinstraße (DÜW), Neustadt an der Weinstraße (NW), and Südliche Weinstraße (SÜW) with the city of Landau (LD); abbreviations stand for the corresponding towns the vineyards were nearby: DH Deidesheim, NK Niederkirchen, KF Kleinfischlingen, WH Wollmesheim, and MH Mühlhofen.

In Germany, in the south of Rhineland Palatinate, 17 different vineyards were selected, with four to five vineyards per *Vitis vinifera* (L.) variety, comprising white (’Riesling’, ’Chardonnay’) and red (’Dornfelder’, ’Pinot Noir’) ones. Vineyards were located between 49°11${}^{\prime }$15.1″ N–49°42${}^{\prime }$86.3″ N and 8°06${}^{\prime }$50.8″ E–8°20${}^{\prime }$97.7″ E (Figure 1) next to the towns Deidesheim (DH), Kleinfischlingen (KF), Mühlhofen (MH), Niederkirchen (NK), and Wollmesheim (WH). Most of the vineyards were located next to Wollmesheim comprising all used *Vitis vinifera* (L.) varieties, with ’Riesling’ only being located there.

Plots of ’Chardonnay’ were also located in Niederkirchen, as well as Kleinfischlingen. The plot next to Deidesheim was planted with ’Pinot Noir’ and the one in Mühlhofen with ’Dornfelder’. Vine and row spacing in the plots were approximately the same and the greening in the tracks was well-kept. All vineyards were roughly north–south oriented, except one in Deidesheim (DH 01). Additional information can be found in the Table A1.

#### 2.1.2. Sampling and Processing Samples

**Figure 2:**
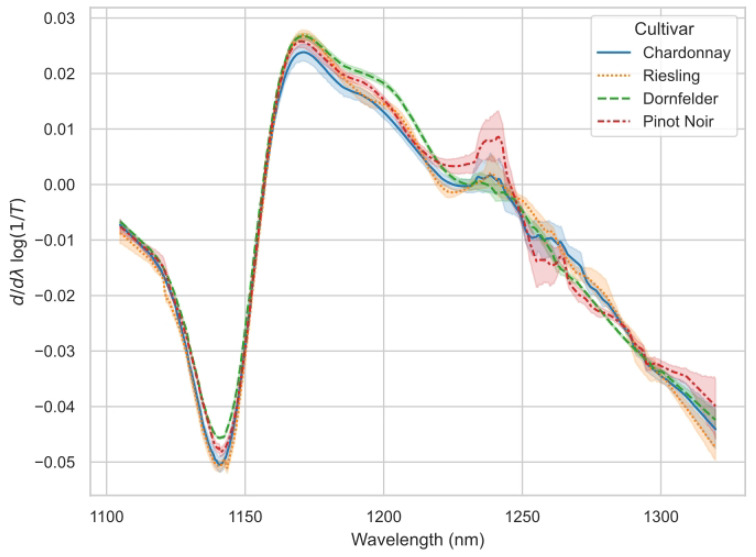
Average recorded and, according to described spectral pretreatments, normalized and filtered transmission spectra of the must in the wavelength range from 1100 nm to 1350 nm, and their corresponding 95% confidence interval (semi-transparent areas) showing the spectral changes between the different samples of the *Vitis vinifera* (L.) cultivars ’Chardonnay’ (blue, solid line), ’Riesling’ (yellow, dotted), ’Dornfelder’ (green, dashed), and ’Pinot Noir’ (red, dotdashed).

Throughout the ripening period from 9 August 2021 to 1 October 2021 (8 time-points), for ’Riesling’ till 14 October (10 timepoints) 2021, 100 berries were taken randomly from each entire vineyard every week. Sampled vines were selected randomly, but evenly distributed over the entire plot. Care was taken to use berries from different positions on the vine, the grape bunch and according to the exposure to sunlight. To gain the must, berries were crushed with a blender (BL 6280, Grundig, Germany) and centrifuged at 20,340× *g* for 10 min (Sigma 6K 15, Sigma Laborzentrifugen GmbH, Osterode am Harz, Germany). Must was then poured through a 100 µm sieve into two 50 mL falcon tubes and used for recording spectral data (Figure 2) and reference values, respectively.

### 2.2. Collection of Reference Values

For the analysis of sugars and acids two laboratory methods were used, (1) HPLC and (2) FTIR.

#### 2.2.1. High-Performance Liquid Chromatography

A subsample of the must was transferred to a 2 mL reaction tube, centrifuged (12,100× *g* for 5 min, Minispin Eppendorf, Hamburg, Germany) and 1:3 diluted using double distilled water. After mixing and another centrifugation step the dilution was filled into vials and placed in the multisampler (G7167B) of the HPLC apparatus (Agilent 12900Infinity II, Agilent Technologies Inc., Santa Clara, CA, USA). The HPLC system consited of a binary pump (G7120A) and a Rezex ROA-Organic Acid H^+^ ion exclusion column (300 mm × 7.8 mm, 8 µm) protected by a security guard Carbo-H^+^ column (Phenomenex Inc., Torrance, CA, USA) kept in a column oven (G7116B) at 75 °C was used for separation. Detection of malic acid and tartaric acid was conducted for diode array detector (G7117B) at 210.4 nm. The sugars glucose and fructose were detected using a refractive index detector (G1362A) kept at 50 °C. Then, 5 µL of each sample was injected and analyzed in a 16.5 min run under an aqueous phase of 0.4 mM sulfuric acid with a flow rate of 0.6 mL/min. As multi-component standard a four-stage dilution series was created containing 1.5 g/L to 90 g/L fructose and glucose and 0.15 g/L to 9 g/L malic acid and tartaric acid, respectively. Analysis of data was performed with Agilent OpenLab Chemstation software (Agilent Technologies, Santa Clara, CA, USA).

If the recording of a spectrum had to be repeated later than on the day of sampling, the levels of the target substances were determined again using HPLC.

#### 2.2.2. Fourier-Transform Infrared Spectroscopy

FTIR is a rapid and commonly used spectroscopic method for the measurement of a wide range of wine and must parameters. Therefore, reference values for the sugars and acids content were also recorded using FTIR (WineScan SO_2_, Foss, Denmark) on the same day when the berries were sampled and the respective must was gained. The system was coupled with an ASX-260 auto sampler (Teledyne CETAC, Omaha, NE, USA). Automatically 10 ml of the centrifuged grape must was injected and measured in duplicate. Values for glucose, fructose, malic acid, and tartaric acid (in g/L) were gained, as well as for density (g/mL), pH, total acidity (g/L), volatile acids (g/L), gluconic acid (g/L), ethanol (%), glycerol (g/L), ammonia (mg/L), and alpha amino nitrogen (mg/L). For the quantification of these parameters, the default wavelength range of the system was used. Calibration was verified against classical detection methods and in inter-laboratory proficiency tests every year.

### 2.3. NIR Apparatus and Acquisition of Spectral Data

The samples were examined using Fraunhofer IOSB *SmartSpectrometer* system [13] using a SpectralEngines spectrometer. The NIRONE Sensor S 1.4 detects wavelengths between 1100 nm and 1350 nm with a resolution of 12–16 nm using a single element InGaAs detector and a Fabry–Pérot interferometer for optical filtering. According to the manufacturer, this sensor has a signal-to-noise ratio of 15,000. A halogen lamp HL-2000-HP (Ocean Optics, Orlando, FL, USA) was used as light source which has a wide spectral range from 360 nm to 2400 nm.

The samples were inserted into a 1/4${}^{\prime \prime }$ flow cell (Avantes, Apeldoorn, The Netherlands) extended with a hose at the end of which a tap was attached, to hold the sample stable. For accurate measurement, the optical path (5 mm) was perpendicular to the flow cell and is accordingly as long as the diameter. A step-index fiber optic cable with a diameter of 365 µm and a numerical aperture of 0.22 (Thorlabs, Newton, NJ, USA) was used to connect light source, flow cell and spectrometer. This enabled the conducive use of the dynamic spectrometers and prevents overloading.

**Figure 3:**
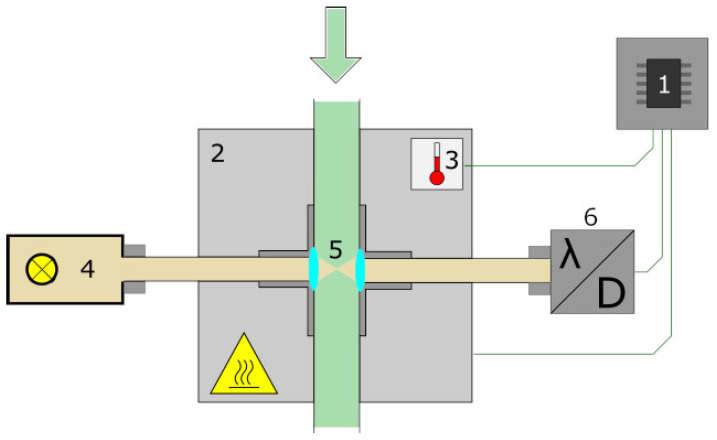
Schematic structure of the miniaturized prototype apparatus: the proportional-integral-derivative (PID) controller (1) regulated the Peltier element (2) depending on the sample temperature (3) and triggered / read out the sensor (6); optical path from left to right (4–6): lamp (4), tube (5), and sensor (6), coupled by fibers.

Due to hydrogen bonding, the sample temperature has an impact on the spectral data. To decrease this influence during harvest, all samples must be measured at precisely defined temperatures for reference. Therefore, each sample had to go through a temperature profile and was measured after reaching specific points. The flow cell mentioned above were attached to a Peltier element with thermal adhesive. The advantage of Peltier elements are that the surface can be heated and cooled by changing the direction of the electric current. The DT-AR-075-24 (European Thermodynamics, Kibworth Harcourt, UK) fits our need and has a maximum cooling power of 74.5 W and an operating range from −10 °C to 47 °C, depending on ambient temperature. To improve the heating process, a fan was located on the opposite side. A classical Pt100 is mounted near the edge of Peltiers surface for temperature measurement. Experiments have shown that the temperature at this position is equal to the temperature of the sample in the flow cell. With this configuration, the temperature can be controlled via a proportional-integral-derivative (PID) controller. However, due to non-linearities of the Peltier element, a classically configured PID controller is not suitable for this task. Therefore, the controller has a non-linear I component that increases faster as temperature is not changing and time is progressing. The setup is shown in Figure 3. Final size of the prototype NIR apparatus was 320 mm × 280 mm × 265 mm.

For temperature reference, each sample is measured at 10 °C to 18 °C in 4 degree increments. Before starting a series of measurements, the system must be referenced with water and 20 °C.

### 2.4. Spectral Processing and Statistical Analysis

The NIR spectra were pre-processed by reducing multiplicative scattering effects using standard normal variates. For smoothing and de-trending of the spectra a Savitzky–Golay filter with 7 points and a polynomial of order 2 in combination with the first derivative was used. Analysis of spectral data and modelling was performed using *spectraltoolbox* framework from Fraunhofer IOSB based on Python 3.8.

Partial least square regression was used to calibrate sugars and acids from near-infrared (NIR) spectra. The number of required latent variables was determined as 6. The PLSR calibration model was validated, using leave-one-out cross-validation. Therefore, the root mean square error of prediction (RMSEP) and R${}^{2}$ are calculated based on samples that are not used for optimization.

Results were visualized using R (Version 4.1.0) [14] and the packages ggplot2 [15], ggpubr [16], and sf [17].

## 3. Results

### 3.1. Reference Data

Data of sugars and acids content of grape must were collected throughout the ripening period at 8 to 10 time points. The dataset for the calculation of the models consisted of the values of the respective six last sampling time points for the different varieties. The amounts and the variances of the ingredients in these given moments can be found in Table A4.

Modelling was performed using reference values gained from HPLC. These data included values in minimum and maximum of 27.63 g/L to 112.19 g/L fructose, 32.37 g/L to 111.70 g/L glucose, 4.54 g/L to 24.38 g/L malic acid, and 5.06 g/L to 12.49 g/L tartaric acid (Table 1). The median limits of quantification (LOQ) over the whole measurement period were 3.34 g/L and 3.85 g/L for fructose and glucose, as well as 0.59 g/L and 0.95 g/L for malic acid and tartaric acid, and could be determined using the calibration measurements according to the guideline on validation of analytical procedures from the International Council on Harmonization.

**Figure 4:**
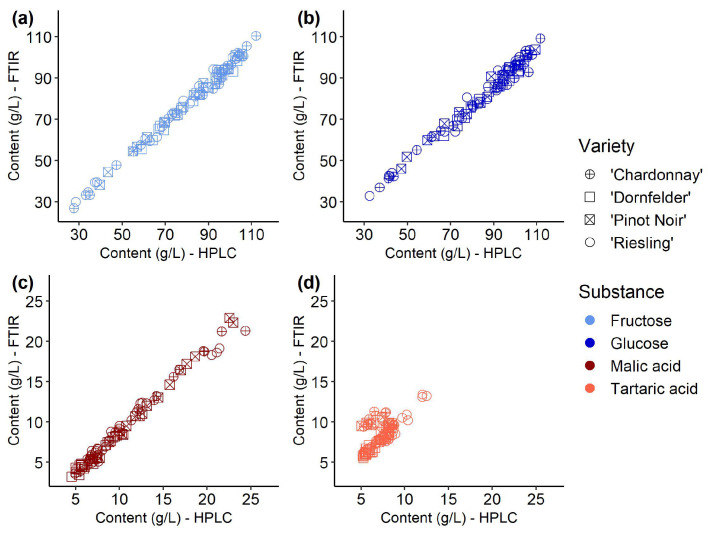
Linear regression of the contents of (**a**) fructose (light blue) (*r*^2^ = 0.99, RMSE = 3.38), (**b**) glucose (dark blue) (*r*^2^ = 0.98, RMSE = 4.83), (**c**) malic acid (dark red) (*r*^2^ = 0.99, RMSE = 1.37), and (**d**) tartaric acid (light red) (*r*^2^ = 0.52, RMSE = 1.66) in reference must samples (n = 87) of four *Vitis vinifera* (L.) varieties, measured using high-performance liquid chromatography (HPLC) and Fourier-transform infrared spectroscopy (FTIR) and the respective root mean square errors (RMSE) and determination coefficients (*r*^2^).

Comparing the reference values measured using HPLC with one of the common standard methods for the determination of ingredients, FTIR, using simple linear regression (see Figure 4) revealed root mean square errors (RMSE) of 3.38 g/L and 4.84 g/L (*r*^2^ = 0.99) for the sugars fructose and glucose, respectively. For the acids, values measured with both methods correlated better for malic acid (RMSE = 1.25 g/L, *r*^2^ = 0.99) than for tartaric acid (RMSE = 0.87 g/L, *r*^2^ = 0.96).

### 3.2. Spectra and Modelling

**Figure 5:**
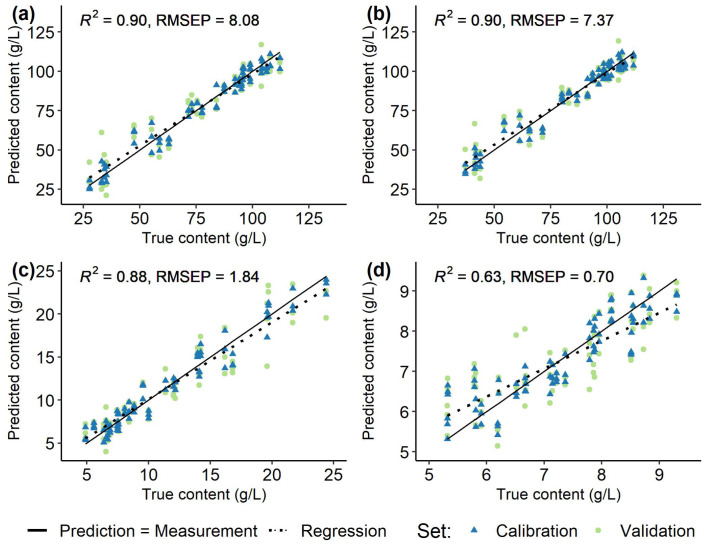
Regression of predicted and measured (true) fructose (**a**), glucose (**b**), malic acid (**c**), and tartaric acid (**d**) contents (g/L) in 100 berries samples from *Vitis vinifera* (L.) cv. ’Chardonnay’ used for calibration (blue) and validation (green) of the models and the respective root mean square errors of prediction (RMSEP) and determination coefficients (*R*^2^) of the validation set. Solid line (Prediction = Measurement) indicates ideal prediction, dashed line (Regression) is a regression of the validation data.

**Figure 6:**
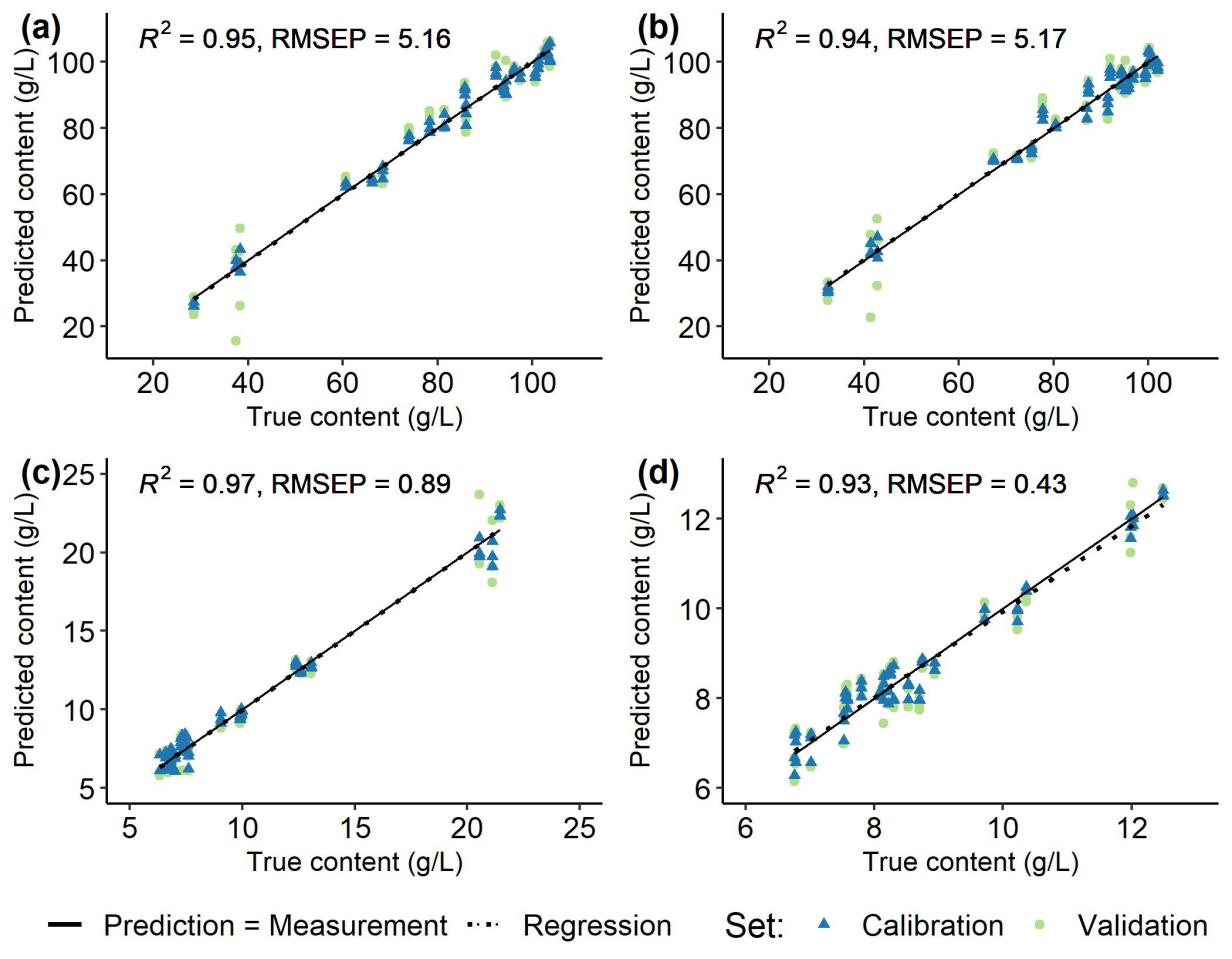
Regression of predicted and measured (true) fructose (**a**), glucose (**b**), malic acid (**c**), and tartaric acid (**d**) contents (g/L) in 100 berries samples from *Vitis vinifera* (L.) cv. ’Riesling’ used for calibration (blue, triangle) and validation (green, circle) of the models and the respective root mean square errors of prediction (RMSEP) and determination coefficients (*R*^2^) of the validation set. Solid line (Prediction = Measurement) indicates ideal prediction, dashed line (Regression) is a regression of the validation data.

**Figure 7:**
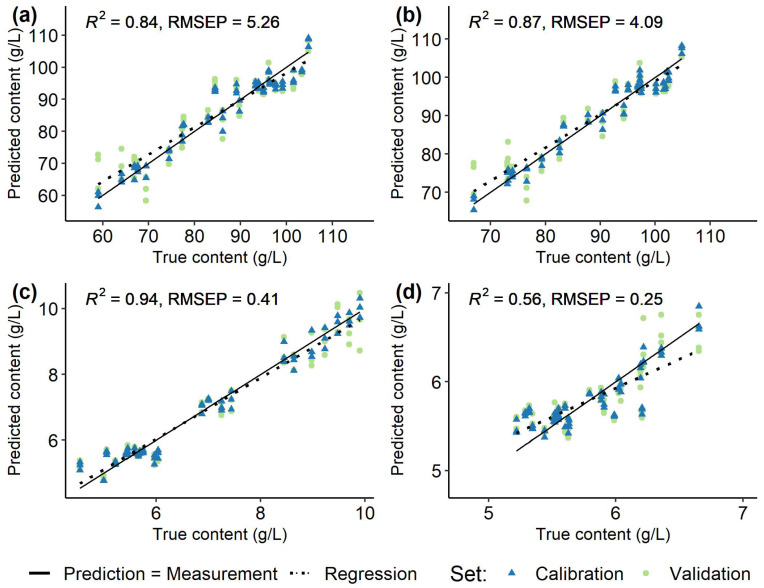
Regression of predicted and measured (true) fructose (**a**), glucose (**b**), malic acid (**c**) and tartaric acid (**d**) contents (g/L) in 100 berries samples from *Vitis vinifera* (L.) cv. ’Dornfelder’ used for calibration (blue, triangle) and validation (green, circle) of the models and the respective root mean square errors of prediction (RMSEP) and determination coefficients (*R*^2^) of the validation set. Solid line (Prediction = Measurement) indicates ideal prediction, dashed line (Regression) is a regression of the validation data.

**Figure 8:**
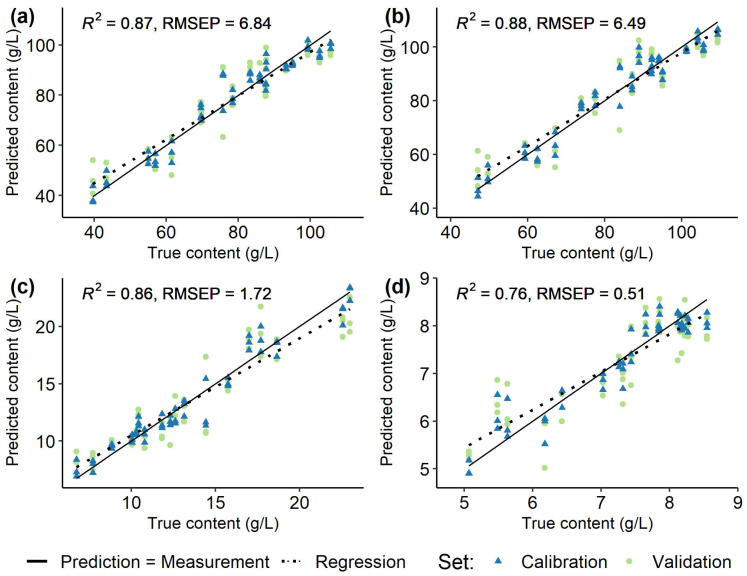
Regression of predicted and measured (true) fructose (**a**), glucose (**b**), malic acid (**c**), and tartaric acid (**d**) contents (g/L) in 100 berries samples from *Vitis vinifera* (L.) cv. ’Pinot Noir’ used for calibration (blue, triangle) and validation (green, circle) of the models and the respective root mean square errors of prediction (RMSEP) and determination coefficients (*R*^2^) of the validation set. Solid line (Prediction = Measurement) indicates ideal prediction, dashed line (Regression) is a regression of the validation data.

Spectral data recorded at the first few time points represented outliers, which had to be excluded due to insufficient referencing with the water blank. Average recorded, normalized, and filtered spectra for each *Vitis vinifera* (L.) cultivar used for modelling are shown in Figure 2. Models were created individually for each grapevine variety (Figure 5, Figure 6, Figure 7 and Figure 8) using the respective reference values gained with HPLC. The resulting different regression coefficients (*R*^2^) and root mean square errors of prediction (RMSEP) are summarised in Table 1.

On average, for red and white *Vitis vinifera* (L.) varieties quality of prediction was comparable. Reliable results were obtained for the sugars that were well predicted by the models, with the RMSEP ranging from 6.86% to 11.49% of the ingredient variance (RMSEP from 4.09 to 8.08 g/L, *R*^2^ 0.84–0.95). For the acids, the results were mixed and the RMSEP (0.25–1.84 g/L, *R*^2^ 0.63–0.97) was 5.89% to 17.57% of the respective value range. Over all the must samples from ’Riesling’ (Figure 6) and ’Chardonnay’ (Figure 5) performed best and were followed by the predictions for ’Dornfelder’ (Figure 7) and ’Pinot Noir’ (Figure 8). The accuracy of the predictions for the two sugars were very similar within and between the respective varieties. The forecasts of the malic acid contents were comparable to the sugars, too. The tartaric acid was well predictable for the variety ’Riesling’ and, in an lesser extent, for the variety ’Pinot Noir’ (see Table 1).

A comparison of the NIR predictions at 14 °C with the results from the FTIR and HPLC measurements for the must of the 100-berries samples revealed excellent predictions for the two sugars and malic acid in all varieties. Tartaric acid prediction was only possible for the variety ’Riesling’, as the HPLC values did not correlate well with the values gained with FTIR for the varieties ’Pinot Noir’, ’Chardonnay’, and ’Dornfelder’ (see Table A3) due to delays in spectral data acquisition.

## 4. Discussion

A fast and reliable method for the quantification of substances that determine quality in viticulture is the FTIR. This method is based on the indirect determination of compounds using infrared light over broad spectral ranges in the mid-infrared light. Its calibration with the reference methods of the OIV (International Organisation of Vine and Wine) and the advantages of measuring with it led to its widespread use in viticulture. In wineries, any new technology will have to compete with it.

Another highly precise method to quantify non-volatile compounds is the HPLC. It is restricted to laboratories and not used in wineries due to its sensitivity and the knowledge required. Both methods were used for the reference measurements in this study. The participation of the methods in a round robin test with several other participating laboratories (data not shown) confirmed their correct measurements. Comparing HPLC and FTIR showed high correlation coefficients and low root mean square errors (see Figure 4) for the two sugars and malic acid. The so gained high-quality dataset provided the opportunity to calibrate the miniaturized prototype sensor system for estimating sugars and acids contents with an accuracy at the laboratory level.

Adequate and reliable models for all varieties could be built for the both sugars glucose and fructose, as well as for the malic acid with comparable prediction accuracies (see Table 1). RMSEPs were lowest for all substances in ’Riesling’ and, except for tartaric acid, *R*^2^ values also showed good prediction accuracies for the other three varieties. Due to the high dependence of the RMSEP and *R*^2^ on the span of values, a direct comparison of the models for the respective varieties is difficult. However, with regard to the range of values, predictions for ’Riesling’ were best for all substances. The RMSEP accounts for only 5.89% to 7.50% of the total variance in this variety. These results are followed by the predictions for ’Chardonnay’, ’Dornfelder’, and ’Pinot Noir’.

The used spectrometer was chosen due to its wavelength range. Krause et al. [13] showed that the wavelength ranges from 1100 nm to 1160 nm and from 1300 nm to 1350 nm could be attributed to acids and sugars, respectively, in red grapevine varieties. Additionally, spectral influences of temperature were minimized in this experiment by recording the spectra of the samples at defined temperatures to calculate the models. Moreover, sugar concentration has a similar effect on the spectra [18]. Ripe berries contain 22% to 25% sugar, so their concentrations are near the tipping point between structure-breaking and structure-making effects of the different sugar concentrations. Variance in the dataset could not been eliminated, but this could have additionally changed differences of the RMSEPs in different content ranges. Therefore, lower variations and lower RMSEPs could be calculated for must of ripe berries. Thus, the predictions of the sugars at higher, as well as malic acid at lower concentrations are best (see Table A2).

In the literature, sugar content in grape must is often measured with the total soluble solids content and great predictions were possible using NIRS [19]. Investigations of fruit composition using NIR above 1100 nm and, in particular, for measuring in transmission mode are rare. For the respective sensors used by Fernández-Novales et al. [12] (700–1060 nm) and González-Caballero et al. [11] (380–1650 nm) calibration, as well as validation for sugar contents worked well, with coefficients of determination (*R*^2^) of cross-validation of 0.98 and 0.86, respectively. However, for must of ripe grapevine berries standard errors of 0.47 °Brix to 1.82 °Brix would lead to measurement uncertainties of the equivalent of circa ±8 g/L to ±20 g/L sugar. In comparison, the correlation coefficients and prediction accuracies in the presented study were excellent (see Table 1).

However, despite good predictions for the sugar contents so far, it has hardly been possible until now to calibrate the acidity well using NIRS, let alone predict it. [19]

In the study of Fernández-Novales et al. [12] it was possible to successfully calibrate pH and titratable acidity with *R*^2^ values of 0.81 and 0.76, respectively. However, calibration failed with correlation coefficients below 0.30. González-Caballero et al. [11] were able to achieve slightly better results for the validation of pH values using a higher wavelength range. Additionally, attempts were made to predict individual acids, as was performed in the presented study. Although calibration for malic acid was possible (*R*^2^ = 0.77), validation failed due to the low correlation coefficient in the study of González-Caballero et al. [11]. The current results confirm that modelling was more successful for malic acid. In fact, in the presented study it was predictable in an equal extent like the sugars glucose and fructose for all varieties.

Tartaric acid was also not predictable in the study of González-Caballero et al. [11], as the correlation coefficients of calibration and cross-validation were near zero. In the actual study, tartaric acid, except for the variety ’Riesling’ (RMSEP = 0.43 g/L, *R*^2^ = 0.93), had the lowest prediction accuracies with RMSEPs ranging from 0.25 g/L to 0.70 g/L and *R*^2^ from 0.56 to 0.76 for the other three varieties. Since the range of contents of this acid are much smaller in these varieties compared to that for ’Riesling’, it is very likely that the poor prediction accuracy is evident from the dataset. The majority of tartaric acid in the berries can probably be diluted by their growth from véraison onwards. In the larger berries, its content drops particularly sharply [20], resulting in only a small range of values as soon as the berries begin to increase in volume. Since the ’Riesling’ has the smallest berries and a late ripening start compared to the other varieties, there was a wider range of values. With the help of these data, however, it could be shown that the prediction of tartaric acid in biological samples with complex matrices, such as grapevine berries, is quite possible if the mathematical basis is given. For this a larger value range should be considered, possibly by collecting more unripe and small berries. These could then be used to compute models for better prediction results. In addition, the measurements of the ingredients with both reference methods should be carried out at the same time as the recording of spectral data. Since tartaric acid contents decreases rapidly when the sample is stored, accuracy of the NIR predictions compared to FTIR reference values were lowered. Chemometric models were unaffected by this, since they were calculated using HPLC values recollected when the spectral data were acquired on a different day. However, delays in spectral data acquisition led to a reduction in the already small range of values.

For wines, it has already been shown that NIRS can be used to distinguish the origin of wine over large and small distances. It was assumed that climatic differences, microclimatic influences and differences in soil composition and topology could be the cause for this possibility of differentiation [21,22,23]. This could even be shown for grapevine berries in the study of Arana et al. [24], in which berries of two white varieties (’Chardonnay’ and ’Viura’) could be assigned to their closely spaced origins with the help of NIRS. Therefore, the origin of the musts could also be a factor influencing the evaluation, since ’Riesling’ plots that achieved the best results, were located close to each other. For all other varieties, the plots were further apart. In particular, the ’Chardonnay’ and ’Pinot Noir’ datasets each contained an area in the northern Rhine plain where slightly contrasting growth conditions prevail.

In addition to the measurements carried out, a promising attempt was made to implement the sensor on a grape harvesting machine. For calibration, spectra at different temperature steps were used for each sample. During harvest, each sample would then only have to be measured once, with the recorded temperature contributing as additional information to the prediction accuracy. A device for sampling, a filter unit (20 µm) and an identically constructed prototype sensor system were set up on a harvester. Automatic acquisition of a low number of spectra was possible, but further improvements are needed. The system has to be optimized for high-throughput spectra collection and an adequate sample flow must be ensured on the grape harvesting machine. Being able to automatically estimate the average quality of the grapes in the harvester’s tank would close the large gap between guesswork and a true estimate. If the method is established for the grape harvesters, this would help save time and money during the busiest part of the year and help the viticulturists making decisions. It will be a big step towards digitization in viticulture and is a basis for the development of complex systems for selective harvest, or the identification of target areas for quality-improving methods, such as fertilization and yield adjustment. A future expansion of the system with additional sensors and new calibrations to record more quality-determining properties will be possible and probable. If this sensor is coupled with a non-destructive, near-infrared handheld device measuring ripeness in the field by winegrowers and making these data available, the cooperatives will have more planning security and the current successes of viticulture in the region could be tracked and possibly also used scientifically.

## 5. Conclusions

Taking all data and results into account, it can be concluded that the models developed are well suited for predicting the sugars and acids contents in must of different *Vitis vinifera* (L.) varieties with a miniaturized near-infrared sensor in the wavelength range of 1100 nm to 1350 nm. A semi-automated prototype near-infrared spectrometer with a temperature control and a flow cell was built, with which the must samples were measured at different temperatures. Using corresponding reference measurements and chemometric methods models for the prediction of quality determining ingredients could be calculated and successfully applied. The accuracies achieved for high sugar and low acid contents, which are prevalent in ripe berries, predestines this technique for high-quality estimates during harvest. Further investigations should be addressed to the implementation of the apparatus on a grape harvester for automated determination of the major quality traits during harvest. Since the grapes in the vineyard ripen unevenly, no exact prediction of harvested grape quality can be made and until now there is no commercial available device that can be incorporated on the grape harvester during the harvesting process. This could simplify and accelerate processes in viticulture, advance digitalization, and increase sustainability through planning security. 

## Figures and Tables

**Table 1:** Value range (minimum and maximum contents) of the analyzed ingredients in the different *Vitis vinifera* (L.) varieties from the dataset used for model calculations and their respective root mean square errors of prediction (RMSEP), determination coefficients (*R*^2^), as well as the number of the must samples (n_samples_) and used corresponding spectra (n_spectra_) recorded from 10 °C to 18 °C in 4 °C increments.

Variety	Substance	Value Range			RMSEP	*R* ^2^
(n_sample_/n_spectra_)		(g/L)			(g/L)	
’Chardonnay’	Fructose	27.63	–	112.19	8.08	0.90
(26/78)	Glucose	37.12	–	111.70	7.37	0.90
	Malic acid	4.89	–	24.38	1.84	0.88
	Tartaric acid	5.32	–	9.30	0.70	0.63
’Riesling’	Fructose	28.49	–	103.68	5.16	0.95
(22/66)	Glucose	32.37	–	101.87	5.17	0.94
	Malic acid	6.34	–	21.44	0.89	0.97
	Tartaric acid	6.76	–	12.49	0.43	0.93
’Dornfelder’	Fructose	59.02	–	104.79	5.26	0.84
(23/69)	Glucose	66.93	–	104.82	4.09	0.87
	Malic acid	4.54	–	9.90	0.41	0.94
	Tartaric acid	5.22	–	6.65	0.25	0.56
’Pinot Noir’	Fructose	39.59	–	105.64	6.84	0.87
(18/54)	Glucose	47.09	–	109.32	6.49	0.88
	Malic acid	6.73	–	23.00	1.72	0.86
	Tartaric acid	5.06	–	8.55	0.51	0.76

## Data Availability

The data presented in this study are available on request from the corresponding author.

## Appendix A

**Table A1:** Table of the vineyards in Rhineland Palatinate (Germany) including the abbreviations used, the planted variety, as well as the year of planting, the applied farming practise (Org: organic, Con: conventional), the corresponding planted rootstocks, and the orientation of the plots.

Abbreviation	*Vitis vinifera*	Planting	Farming	Root-	Orientation
	Variety	Year	Practice	Stock	
DH 01	Pinot Noir	2000	Org	26 G	W-O
KF 02	Chardonnay	2016	Con	SO 4	N-S
MH 01	Dornfelder	1997	Org	5 C	N-S
NK 01	Chardonnay	2010	Org	SO 4	N-S
WH 01	Riesling	2005	Org	5 C	N-S
WH 02	Chardonnay	2001	Org	SO 4	N-S
WH 03	Dornfelder	1997	Org	5 C	N-S
WH 04	Riesling	1991	Con	5 C	N-S
WH 05	Pinot Noir	1990	Org	SO 4	N-S
WH 06	Dornfelder	2002	Con	125 AA	N-S
WH 07	Chardonnay	2005	Con	SO 4	N-S
WH 08	Riesling	1991	Org	Binova	N-S
WH 09	Pinot Noir	2004	Con	125 AA	N-S
WH 10	Riesling	2006	Con	5 C	N-S
WH 11	Chardonnay	1990	Con	SO 4	N-S
WH 12	Dornfelder	2004	Con	125 AA	N-S
WH 13	Pinot Noir	2001	Con	SO 4	N-S

**Table A2:** Root mean square errors of prediction (RMSEP) from the validation set for the ranges below (RMSEP _low_) and above (RMSEP _high_) the median content of the four substances and their corresponding number of samples (n).

*Vitis vinifera*	Substance	Median Content	RMSEP _low_	n _low_	RMSEP _high_	n _high_
variety		(g/L)	(g/L)		(g/L)	
Chardonnay	Fructose	85.67	9.89	39	5.73	39
	Glucose	92.43	9.17	39	4.96	39
	Malic acid	9.76	1.3	39	2.25	39
	Tartaric acid	7.29	0.73	39	0.66	39
Riesling	Fructose	89.12	6.31	33	3.66	33
	Glucose	91.66	6.33	33	3.65	33
	Malic acid	7.57	0.64	33	1.08	33
	Tartaric acid	8.27	0.42	33	0.44	33
Dornfelder	Fructose	89.07	6.29	36	3.98	36
	Glucose	94.25	4.84	36	3.21	36
	Malic acid	6.04	0.38	36	0.45	36
	Tartaric acid	5.63	0.22	36	0.27	36
Pinot Noir	Fructose	80.81	7.27	27	6.37	27
	Glucose	87.95	6.88	27	6.08	27
	Malic acid	12.46	1.26	27	2.08	27
	Tartaric acid	7.55	0.57	27	0.43	27

**Table A3:** Root mean square errors (RMSE) and correlation coefficients (*r*^2^) of the comparisons between NIR predictions and FTIR measurements (RMSE / *r*^2^ _F_), NIR predictions and HPLC measurements (RMSE / *r*^2^ _H_) and between HPLC and FTIR measurements (RMSE / *r*^2^ _HF_); NIR predictions for spectra recorded at 14 °C were used for calculation.

*Vitis vinifera*	Substance	RMSE _F_	*r*^2^ _F_	RMSE _H_	*r*^2^ _H_	RMSE _HF_	*r*^2^ _HF_	n
Variety		(g/L)		(g/L)		(g/L)		
Chardonnay	Fructose	10.11	0.88	9.89	0.88	3.65	0.99	26
	Glucose	9.50	0.88	8.63	0.89	5.47	0.99	26
	Malic acid	2.43	0.86	2.01	0.88	1.38	0.99	26
	Tartaric acid	2.55	−0.04	0.60	0.70	2.36	−0.02	26
Riesling	Fructose	3.29	0.98	3.54	0.98	2.73	0.99	22
	Glucose	4.21	0.97	4.03	0.96	4.04	0.98	22
	Malic acid	1.41	0.97	0.71	0.98	1.36	0.98	22
	Tartaric acid	0.69	0.92	0.39	0.95	0.64	0.94	22
Dornfelder	Fructose	6.03	0.89	4.60	0.88	4.06	0.99	23
	Glucose	6.59	0.88	3.67	0.90	5.27	0.97	23
	Malic acid	1.35	0.90	0.42	0.95	1.42	0.95	23
	Tartaric acid	0.63	0.09	0.22	0.63	0.60	0.29	23
Pinot Noir	Fructose	7.19	0.87	6.55	0.88	2.73	0.99	18
	Glucose	7.26	0.88	5.73	0.90	4.28	0.98	18
	Malic acid	2.03	0.87	1.67	0.86	1.32	0.99	18
	Tartaric acid	2.28	−0.03	0.5	0.76	2.23	0	18

**Table A4:** Variation in the contents of fructose, glucose, malic acid, and tartaric acid in g / L over different timepoints (Day), additionally shown are the respective numbers of the spectra (n) used for modelling, the minimum and maximum (min–max), the median, as well as the respective standard deviation of the contents (sd); depicted are the reference values measured with RID/DAD HPLC used for chemometry; sampling took place weekly, the timepoints T3–T8 correspond to the weeks from 23 August 2021–29 August 2021 (T3) to 27 September 2021–10 October 2021 (T8), for Riesling further samples were taken until 14 October 2021 (T9, T10).

Timepoint (n)		Fructose (g / L)	Glucose (g / L)	Malic Acid (g / L)	Tartaric Acid (g / L)
’Chardonnay’					
T3 (4)	min–max	27.63–35.10	37.12–43.62	19.59–24.38	6.51–7.87
	median (sd)	33.58 (3.03)	41.52 (2.48)	20.70 (2.03)	7.22 (0.65)
T4 (4)	min–max	47.34–62.81	54.35–71.31	13.97–16.82	5.32–6.19
	median (sd)	56.90 (5.93)	63.35 (6.47)	15.20 (1.28)	5.86 (0.33)
T5 (4)	min–max	71.48–83.97	79.90–91.38	9.52–12.16	8.51–8.83
	median (sd)	74.25 (5.03)	81.63 (4.84)	11.69 (1.10)	8.63 (0.14)
T6 (5)	min–max	77.47–95.96	86.61–106.30	6.89–14.10	7.86–9.30
	median (sd)	92.27 (7.01)	96.19 (6.76)	8.42 (2.59)	8.16 (0.54)
T7 (5)	min–max	94.12–106.36	98.33–107.94	5.56–10.00	5.32–8.50
	median (sd)	98.87 (4.36)	100.68 (3.63)	7.50 (1.46)	6.21 (1.17)
T8 (4)	min–max	103.84–112.19	105.02–111.70	4.89–6.68	6.68–7.37
	median (sd)	106.09 (3.49)	106.06 (2.77)	6.46 (0.75)	7.12 (0.26)
’Riesling’					
T5 (3)	min–max	28.49–38.26	32.37–42.81	20.53–21.44	11.99–12.49
	median (sd)	37.39 (4.68)	41.34 (4.90)	21.10 (0.40)	12.02 (0.24)
T6 (3)	min–max	60.63–68.40	67.23–75.31	12.39–13.06	9.72–10.36
	median (sd)	66.17 (3.47)	72.24 (3.53)	12.60 (0.30)	10.22 (0.29)
T7 (4)	min–max	73.99–85.99	77.63–91.37	9.03–9.99	7.58–8.94
	median (sd)	79.85 (4.57)	83.72 (5.64)	9.93 (0.41)	8.39 (0.52)
T8 (4)	min–max	85.69–94.41	87.30–95.76	7.27–7.53	7.55–8.22
	median (sd)	93.83 (3.78)	94.61 (3.53)	7.39 (0.11)	7.97 (0.28)
T9 (4)	min–max	92.24–101.12	91.94–101.87	6.34–7.62	6.76–7.53
	median (sd)	98.93 (3.68)	98.02 (3.83)	6.80 (0.51)	6.89 (0.32)
T10 (4)	min–max	96.16–103.68	94.25–100.39	6.73–6.83	8.10–8.75
	median (sd)	102.77 (3.16)	100.08 (2.69)	6.81 (0.04)	8.50 (0.28)
’Dornfelder’					
T3 (3)	min–max	59.02–69.39	66.93–76.55	9.47–9.90	6.22–6.65
	median (sd)	64.08 (4.49)	73.20 (4.23)	9.70 (0.19)	6.36 (0.19)
T4 (4)	min–max	66.88–77.26	73.07–82.53	8.45–9.23	5.79–6.19
	median (sd)	71.02 (4.62)	76.66 (4.06)	8.81 (0.32)	5.95 (0.16)
T5 (4)	min–max	77.59–89.73	83.29–94.25	6.87–7.44	5.52–5.91
	median (sd)	84.51 (4.66)	89.09 (4.17)	7.13 (0.23)	5.58 (0.16)
T6 (4)	min–max	84.46–95.00	92.75–101.84	5.42–6.04	5.35–5.99
	median (sd)	91.49 (4.38)	99.36 (3.85)	5.72 (0.23)	5.54 (0.26)
T7 (4)	min–max	93.25–99.10	95.06–100.11	5.05–5.66	5.29–6.20
	median (sd)	97.00 (2.25)	97.11 (1.90)	5.52 (0.25)	5.43 (0.39)
T8 (4)	min–max	96.09–104.79	97.16–104.82	4.54–5.96	5.22–6.04
	median (sd)	102.43 (3.44)	102.29 (2.91)	5.11 (0.54)	5.57 (0.31)
’Pinot Noir’					
T3 (1)		39.59	47.09	23	7.44
T4 (4)	min–max	43.37–69.58	49.63–77.49	12.61–22.57	5.06–6.43
	median (sd)	55.90 (9.71)	60.88 (10.45)	17.82 (3.73)	5.90 (0.55)
T5 (4)	min–max	61.44–87.62	67.11–94.97	10.42–17.69	7.65–8.55
	median (sd)	74.05 (10.19)	80.47 (11.39)	14.43 (2.86)	8.03 (0.36)
T6 (4)	min–max	75.74–99.25	83.91–104.12	7.71–14.42	7.26–8.27
	median (sd)	89.59 (9.15)	93.10 (7.51)	11.30 (2.59)	8.15 (0.43)
T7 (3)	min–max	83.24–105.64	92.19–109.32	6.73–11.83	5.48–7.32
	median (sd)	95.01 (9.71)	101.18 (7.42)	10.05 (2.24)	7.03 (0.85)
T8 (2)	min–max	87.68–102.57	88.84–105.61	8.84–10.78	7.86–8.16
	median (sd)	95.12 +(8.16)	97.23 (9.18)	9.81 (1.06)	8.01 (0.16)
